# NK cell defects in X-linked pigmentary reticulate disorder

**DOI:** 10.1172/jci.insight.125688

**Published:** 2019-11-01

**Authors:** Petro Starokadomskyy, Katelynn M. Wilton, Konrad Krzewski, Adam Lopez, Luis Sifuentes-Dominguez, Brittany Overlee, Qing Chen, Ann Ray, Aleksandra Gil-Krzewska, Mary Peterson, Lisa N. Kinch, Luis Rohena, Eyal Grunebaum, Andrew R. Zinn, Nick V. Grishin, Daniel D. Billadeau, Ezra Burstein

**Affiliations:** 1 Department of Internal Medicine, University of Texas Southwestern Medical Center, Dallas, Texas, USA.; 2 Department of Immunology and Department of Biochemistry and Molecular Biology, Mayo Clinic College of Medicine, Mayo Clinic, Rochester, Minnesota, USA.; 3 Receptor Cell Biology Section, Laboratory of Immunogenetics, National Institute of Allergy and Infectious Diseases, NIH, Rockville, Maryland, USA.; 4 Department of Pediatrics, University of Texas Southwestern Medical Center, Dallas, Texas, USA.; 5 Department of Surgery, Tongji University affiliated Tongji Hospital, Shanghai, China.; 6 Department of Microbiology, University of Texas Southwestern Medical Center, Dallas, Texas, USA.; 7 Molecular and Cellular Immunology Section, Laboratory of Immunogenetics, National Institute of Allergy and Infectious Diseases, NIH, Rockville, Maryland, USA.; 8 Howard Hughes Medical Institute, University of Texas Southwestern Medical Center, Dallas, Texas, USA.; 9 Division of Genetics, Department of Pediatrics, San Antonio Military Medical Center, San Antonio, Texas, USA.; 10 Division of Immunology and Allergy and Department of Pediatrics, Developmental and Stem Cell Biology Program, Research Institute, Hospital for Sick Children, Toronto, Ontario, Canada.; 11 Eugene McDermott Center for Human Growth and Development,; 12 Department of Biochemistry,; 13 Department of Biophysics, and; 14 Department of Molecular Biology, University of Texas Southwestern Medical Center, Dallas, Texas, USA.

**Keywords:** Infectious disease, Inflammation, Innate immunity, Monogenic diseases, NK cells

## Abstract

X-linked reticulate pigmentary disorder (XLPDR, Mendelian Inheritance in Man #301220) is a rare syndrome characterized by recurrent infections and sterile multiorgan inflammation. The syndrome is caused by an intronic mutation in *POLA1,* the gene encoding the catalytic subunit of DNA polymerase-α (Pol-α), which is responsible for Okazaki fragment synthesis during DNA replication. Reduced *POLA1* expression in this condition triggers spontaneous type I interferon expression, which can be linked to the autoinflammatory manifestations of the disease. However, the history of recurrent infections in this syndrome is as yet unexplained. Here we report that patients with XLPDR have reduced NK cell cytotoxic activity and decreased numbers of NK cells, particularly differentiated, stage V, cells (CD3^–^CD56^dim^). This phenotype is reminiscent of hypomorphic mutations in *MCM4*, which encodes a component of the minichromosome maintenance (MCM) helicase complex that is functionally linked to Pol-α during the DNA replication process. We find that POLA1 deficiency leads to MCM4 depletion and that both can impair NK cell natural cytotoxicity and show that this is due to a defect in lytic granule polarization. Altogether, our study provides mechanistic connections between Pol-α and the MCM complex and demonstrates their relevance in NK cell function.

## Abstract 2

X-linked reticulate pigmentary disorder is associated with functional NK cell defect due to abnormal lytic granule polarization.

## Graphical abstract

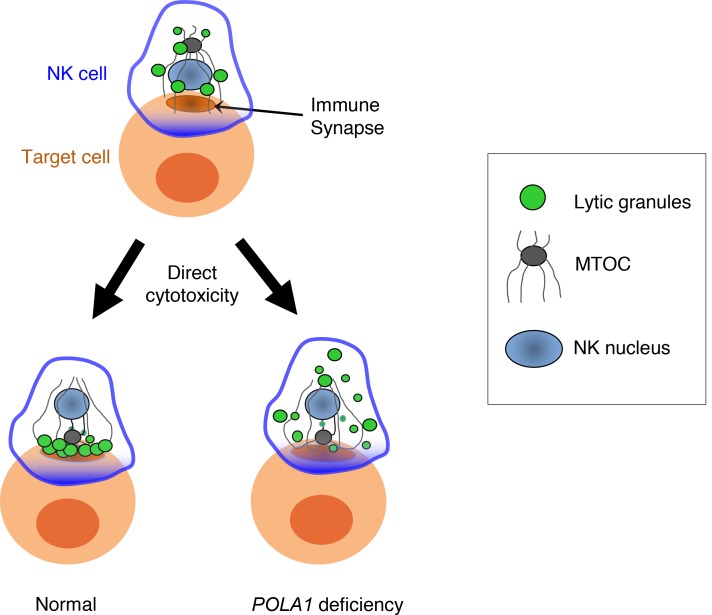

## Introduction

X-linked reticulate pigmentary disorder (XLPDR, Mendelian Inheritance in Man [MIM] #301220) is a rare syndrome that has been reported in only 24 patients worldwide (1–11). It is characterized by constitutional features (unique facial features and hypohidrosis), hyperpigmentation, and sterile multiorgan inflammation involving the eyes (keratitis, corneal scarring, and ultimately blindness), intestine (infantile enterocolitis and jejunal Crohn’s disease), and urinary tract (ureteral and urethral inflammation, scarring, and stricture formation). In addition, most patients suffer from recurrent infections, predominantly in the respiratory tract, resulting in bronchiectasis and respiratory failure (1–3, 9, 10). Previously, we reported that XLPDR is caused by a unique and recurrent intronic mutation in the *POLA1* gene, resulting in missplicing and partial POLA1 protein deficiency (10). *POLA1* encodes the catalytic subunit of DNA polymerase-α (Pol-α), which in vertebrates exists in a stable complex with primase (12). Together with a DNA helicase that unwinds chromosomal DNA, known as the minichromosome maintenance (MCM) complex, Pol-α/primase is responsible for initiating DNA replication. We discovered that POLA1 deficiency in XLPDR is associated with reduced levels of cytosolic RNA/DNA hybrids, which were shown to have immunomodulatory effects, including the modulation of nucleic acid sensors upstream of the type I IFN response. More recently, other mutations in *POLA1* have been reported, which result in severe intrauterine and postnatal growth retardation, intellectual disability, hypogonadism, and in at least 1 case, recurrent serious infections and chronic IFN activation. Interestingly, the facial and cutaneous features of XLPDR are absent in these cases (13).

The immunomodulatory effect of POLA1 deficiency is the likely explanation for the autoinflammatory manifestations of the disease, as we identified in our previous study (10). However, the mechanism behind the immunodeficiency observed in these patients has remained elusive. Here we report that patients with XLPDR have decreased NK cell cytotoxic activity and reduced NK cell counts, particularly a selective reduction in differentiated, stage V, NK cells (CD3^–^CD56^dim^). The reduction in differentiated NK cells is a feature previously described in immunodeficiency 54 (IMD54, MIM #609981), a monogenic disorder due to autosomal recessive mutations in the *MCM4* gene, which encodes a subunit of the MCM complex. This syndrome is characterized by growth retardation, adrenal insufficiency, and a selective NK cell deficiency, affecting most severely differentiated stage V NK cells (14–16). Associated infections in this syndrome include serious and/or recurrent herpes virus infections, including EBV-associated lymphoproliferative disorder (14). In striking similarity to XLPDR, IMD54 can also lead to recurrent infections in the respiratory tract, resulting in bronchiectasis and respiratory failure (17). Evidence presented here links XLPDR to MCM4 deficiency, likely explaining the overlap in clinical features between these 2 genetic syndromes.

## Results

### XLPDR is associated with decreased number and selective cytotoxicity defect of NK cells.

**Figure 1:**
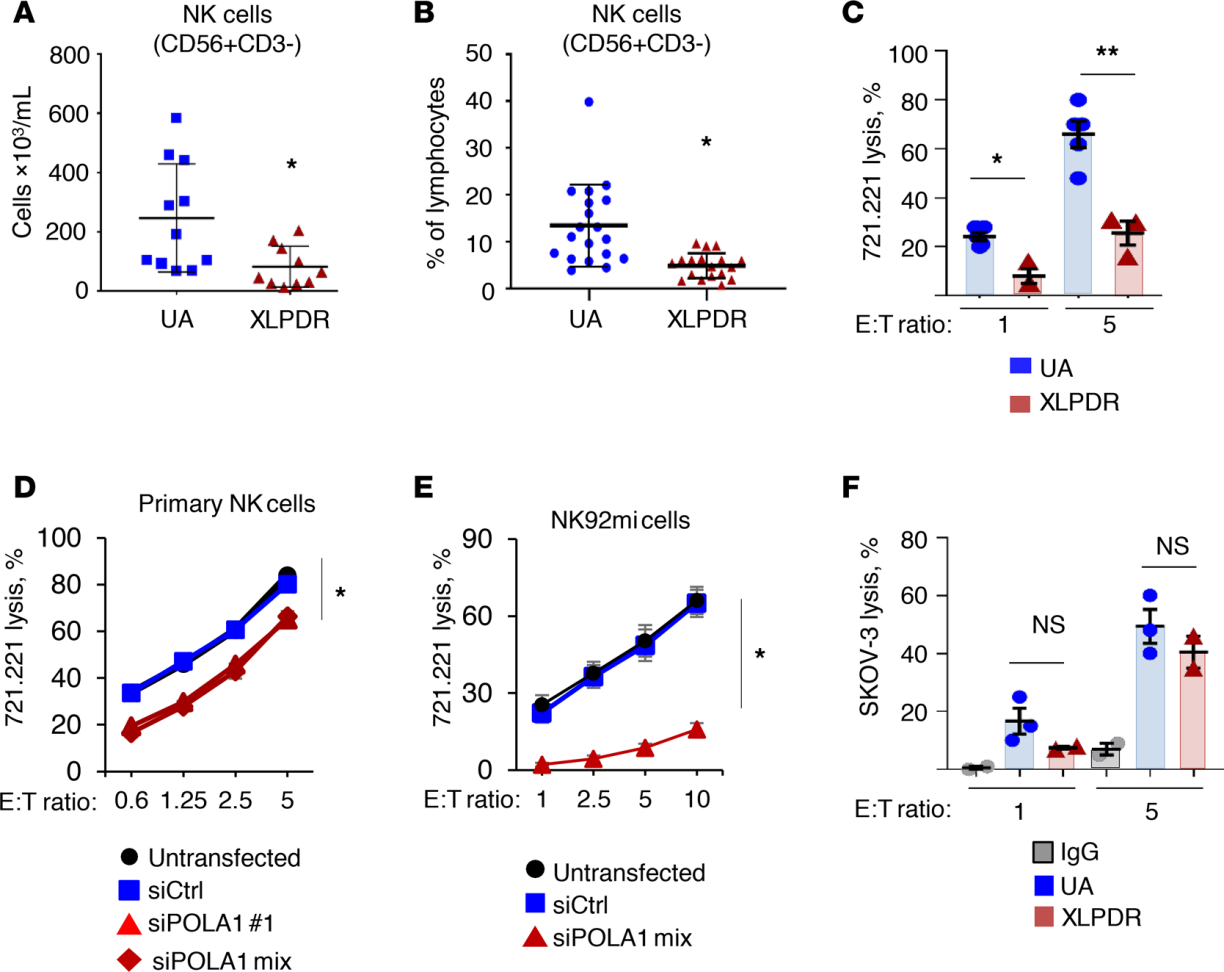
NK cell direct cytotoxicity is affected in XLPDR patients. (**A**) Flow cytometry quantification of NK cells per milliliter in peripheral blood of XLPDR patients (P1–P5) and unaffected individuals (UA4–UA11). Horizontal bars represent the mean; error bars represent the SD. *P < 0.015, Student’s 2-tailed *t* test. Data are the aggregate from up to 3 independent measurements. (**B**) Flow cytometry quantification of NK cells in peripheral blood as a percentage of total lymphocytes. P1–P5 and UA1–UA12 are represented. Horizontal bars represent the mean; error bars represent the SD. *P < 0.0005, Student’s 2-tailed *t* test. Data are the aggregate of 7 independent measurements spanning up to 8 years. (**C**) NK cell direct cytotoxicity against 721.221 target cells was assessed using NK cells from unaffected controls (UA1, UA2, UA13) or XLDPR patients (P1 and P2), using effector/target (E/T) ratios of 1 and 5. Bars represent the mean; error bars represent the SEM. *P < 0.0065, and **P < 0.0001 by 2-way ANOVA. Data are the aggregate of 2 independent experiments. (**D**) NK cell direct cytotoxicity against the 721.221 target cell line was determined over the indicated E/T ratios. Primary NK cells obtained from 5 healthy donors (UA14–UA18) were subjected to *POLA1* silencing using 2 siRNA duplexes or a pool of them. The data are representative of 2 independent experiments; error bars represent the SEM. *P < 0.0001 by 2-factor ANOVA comparing siPOLA1 samples against controls. Data are the aggregate of 5 independent experiments. (**E**) Same as **D** but using the tumor-derived NK cell line NK92mi. The data are representative of 3 independent experiments; error bars represent the SEM. *P < 0.0001 by 2-way ANOVA comparing siPOLA1 mix against control samples. Data are representative of 2 independent experiments. (**F**) Antibody-dependent cell cytotoxicity (ADCC) of NK cells from unaffected controls (UA8–UA10) and XLPDR patients (P1 and P2). SKOV-3 ovarian cancer cells treated with anti–human epidermal growth factor receptor 2 (anti-HER2) antibodies were used as the target cell line; cells incubated with control antibody (IgG) were used as a negative control. Bars represent the mean; error bars represent the SEM. NS, nonsignificant; *P* > 0.1 by 2-factor ANOVA comparing UA to XLPDR groups. Data are from a single experiment.

Despite a history of recurrent infections in XLPDR, prior work has not elucidated the immunological cause for this clinical feature. Previously, we reported that NK cell numbers were in the low end of normal in 2 XLPDR probands (10). Here, we examined this parameter in more detail, with repeated NK cell quantification over a 1- to 6-year period in 5 patients with XLPDR from 3 separate families who reside in the United States and Canada (Supplemental Figure 1A; supplemental material available online with this article; https://doi.org/10.1172/jci.insight.125688DS1). Compared with unaffected individuals without known immune defects, patients with XLPDR had significantly lower NK cell absolute numbers (Figure 1A) and decreased NK cells as a percentage of total lymphocytes (Figure 1B). Using a cutoff of fewer than 50 × 10^3^ cells/mL to define severe NK cell lymphopenia (18), patients with XLPDR were below this threshold 50% of the time, whereas no unaffected control subject fell in this range.

In addition to numerical changes, a clinical analysis of 2 patients with XLPDR revealed decreased NK cell cytotoxic activity against K562 cells, with preserved T cell–mediated cytotoxicity (Table 1). We recapitulated the observed cytotoxic deficiency of primary XLPDR NK cells using a different target cell line, human B lymphoblastoid 721.221 cells (Figure 1C), and reproduced this effect through anti-POLA1 siRNA treatment of primary NK cells or the NK92mi cell line (Figure 1, D and E, and Supplemental Figure 1, B and C). Interestingly, antibody-dependent cell cytotoxicity remained unchanged (Figure 1F), suggesting that these cells have preserved lytic capacity under specific contexts. In agreement with this observation, intracellular staining and flow cytometry indicated that patient-derived NK cells had normal levels of the cytotoxic proteins perforin and granzyme B (Table 2). Moreover, the levels of LAMP1, a late endosome/lysosome and lytic granule-associated protein, were also normal (Supplemental Figure 1D), suggesting that granule biogenesis and expression of key lytic molecules were not affected by POLA1 deficiency.

### POLA1 is linked to MCM4 protein expression.

Gene coexpression analysis can provide insight into functional associations because interacting protein pairs tend to behave similarly across different expression conditions (19). This approach indicated that *POLA1* expression is coregulated with genes encoding other subunits of the Pol-α/primase complex, along with genes in the DNA damage response and cell cycle regulation, including *MCM4* (summary data presented in Table 3, entire analysis available in Supplemental Table 1). POLA1, as the catalytic subunit of Pol-α, works in concert with the MCM and GINS complexes during DNA replication (20). These findings were potentially relevant to this study because mutations in *MCM4* and *GINS1* genes have been reported to cause NK cell deficiency and NK cell maturation defects, with associated predisposition to recurrent infections (14, 15, 21). Therefore, we investigated whether a potential connection between POLA1 and MCM4 could explain the NK cellular phenotypes noted in patients with XLPDR.

**Figure 2:**
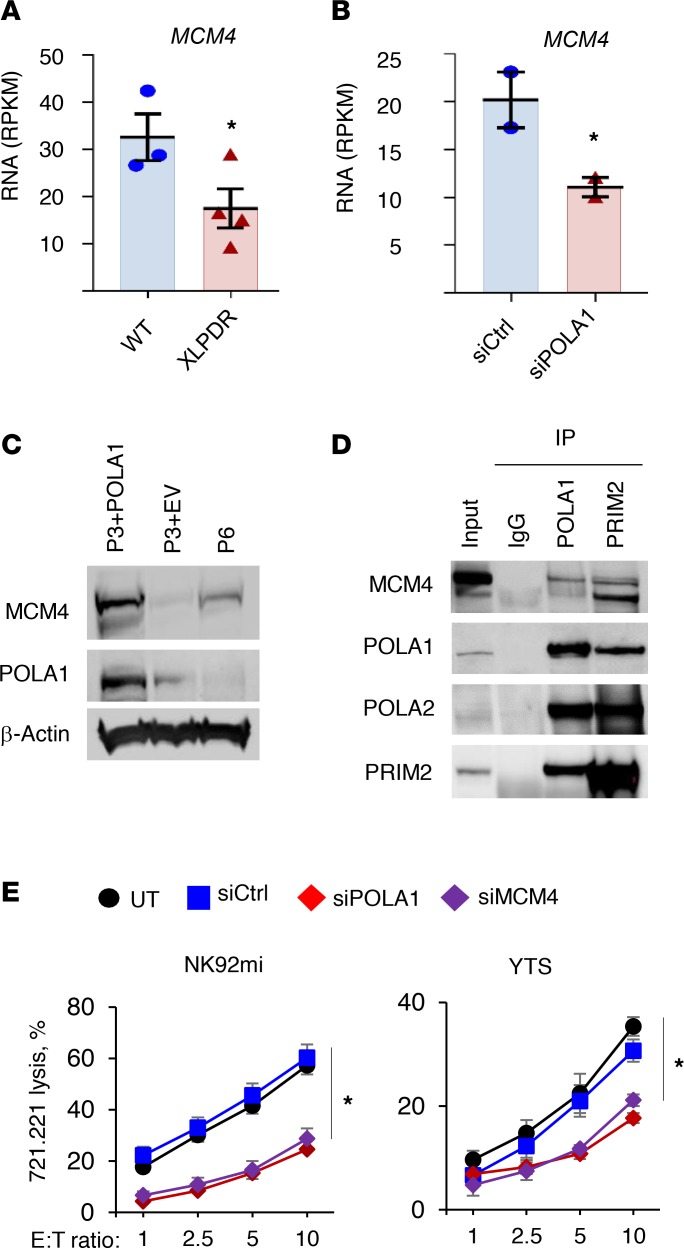
Pol-α/primase and the MCM complex are required for optimal NK cell function. (**A**) Expression of *MCM4* as determined by RNA-Seq analysis in unaffected (UA1) and XLPDR-derived immortalized dermal fibroblasts (XLPDR, P2 and P3). Bars represent the mean; error bars represent the SEM; *P < 0.05 by Student’s 1-tailed *t* test. Data are the average of 3 independent experiments. RPKM, reads per kilobase of transcript per million mapped reads. (**B**) Same as **A**, but comparing *MCM4* expression in UA1 fibroblasts treated with siCtrl or siPOLA1. Bars represent the mean; error bars represent the SEM. *P < 0.05 by Student’s 1-tailed *t* test. Data are the average of 2 independent experiments. (**C**) Expression of the indicated proteins was determined by immunoblotting in immortalized dermal fibroblasts derived from an XLPDR patient (P3 + EV), isogenic “rescued” control line (P3 + POLA1), and a patient (P6) with a recently reported POLA1 mutation (c.328G>A). The Western blot is representative of 2 independent experiments. (**D**) HEK293T cell lysate was subjected to POLA1 or PRIM2 immunoprecipitation and then immunoblotted for MCM4. Nonspecific control antibody (IgG) was used as a negative control. The Western blot is representative of 2 independent experiments. (**E**) NK cell lines NK29mi (left) and YTS (right) were subjected to POLA1 or MCM4 silencing using corresponding siRNA. NK cell direct cytotoxicity against the 721.221 target cell line was determined over the indicated E/T ratios. Data represent the average of 3 experiments. Error bars represent the SEM. *P < 0.0001 by 2-way ANOVA comparing siPOLA1 or siMCM4 against siCtrl in each E/T ratio. Data are from a single experiment.

We began by experimentally confirming the coexpression analysis results. First, as shown in Figure 2A, we noted that previously published RNA-Seq data (Gene Expression Omnibus [GEO] accession number GSE72589, ref. 10) demonstrated decreased *MCM4* mRNA expression in XLPDR-derived dermal fibroblasts (P2 and P3, Supplemental Figure 1A) compared with an unaffected individual (UA1, the father of P2). Similar findings were made when comparing siRNA-mediated silencing of POLA1 to control siRNA in a normal dermal fibroblast line (Figure 2B). Other components of the MCM complex, as well as components of the GINS complex, were similarly downregulated at the mRNA in the same settings (Supplemental Figure 2). When compared with a patient-derived fibroblast line with “rescued” POLA1 expression (P3 + POLA1), the isogenic XLPDR-derived fibroblasts (P3 + empty vector [EV]) displayed decreased MCM4 protein expression (Figure 2C). Furthermore, immunoprecipitation of Pol-α (POLA1 antibody) or primase (PRIM2 antibody) led to recovery of MCM4, indicative of physical interactions between these complexes in vivo and consistent with their tightly linked roles at the DNA replication fork (Figure 2D). Interestingly, MCM4 immunoblots produced 2 bands (Figure 2, C and D), which were both depleted by siRNA (Supplemental Figure 4B), suggesting that they correspond to a long and short isoform of MCM4. This corresponds to previous reports showing that *MCM4* mRNA supports at least 2 alternative start codons and that pathogenic mutations in IMD54 cause a selective loss of the long isoform (14, 15). Interestingly, PRIM2 immunoprecipitation brought down a disproportionate amount of the short MCM4 isoform (Figure 2D), although the significance of this finding is unclear.

In addition, we examined this relationship in dermal fibroblasts from a patient with a recently reported *POLA1* mutation (13) (c.328G>A, denoted as P6, Figure 2C and Supplemental Figure 1A). This mutation is distinct from the recurrent intronic *POLA1* mutation seen in XLPDR (NC_000023.10:g.24744696A>G). The *POLA1* c.328G>A mutation affects the splice donor site of exon 4, resulting in markedly reduced *POLA1* mRNA expression in patient-derived dermal fibroblasts compared with 3 lines derived from normal unrelated males (WT1, WT2, WT3), more severe than that seen in XLPDR-derived cells (Supplemental Figure 3A). At the protein level, P6 fibroblasts displayed profound POLA1 deficiency, and these cells exhibited decreased MCM4 protein expression (Figure 2C). Interestingly, the patient exhibited intellectual disability, severe intrauterine growth delay, and postnatal growth retardation, as well as multiple serious infections (13), features resembling *MCM4* deficiency. Therefore, the patient was screened for adrenal insufficiency (which is part of IMD54) and was found to have normal function. Next, we examined whether the patient had immunological features of XLPDR and found that he had dramatic activation of IFN-stimulated gene (ISG) expression in his blood (Supplemental Figure 3B). Moreover, his dermal fibroblasts displayed baseline ISG elevation and greater sensitivity to nucleic acid ligands compared with control cells (Supplemental Figure 3, C and D). Although P6 had normal NK cell count (217 × 10^3^ cells/mL), cytotoxicity of NK cells and T cells was reduced (Table 1).

Although patients with *MCM4* mutations have markedly reduced numbers of NK cells, the effect of MCM4 deficiency on NK cell direct cytotoxicity has not been previously evaluated. Using siRNA, we assessed whether transient *MCM4* or *POLA1* deficiencies similarly affect direct NK cell cytotoxicity, using different NK cell lines YTS and NK92mi. Indeed, both *MCM4* and *POLA1* silencing similarly decreased direct cytotoxicity (Figure 2E and Supplemental Figure 4A). Altogether, these data implicate both POLA1 and MCM4 in the cytotoxic NK cell response.

### XLPDR is associated with decreased numbers of CD3^–^CD56^dim^ stage V NK cells.

**Figure 3:**
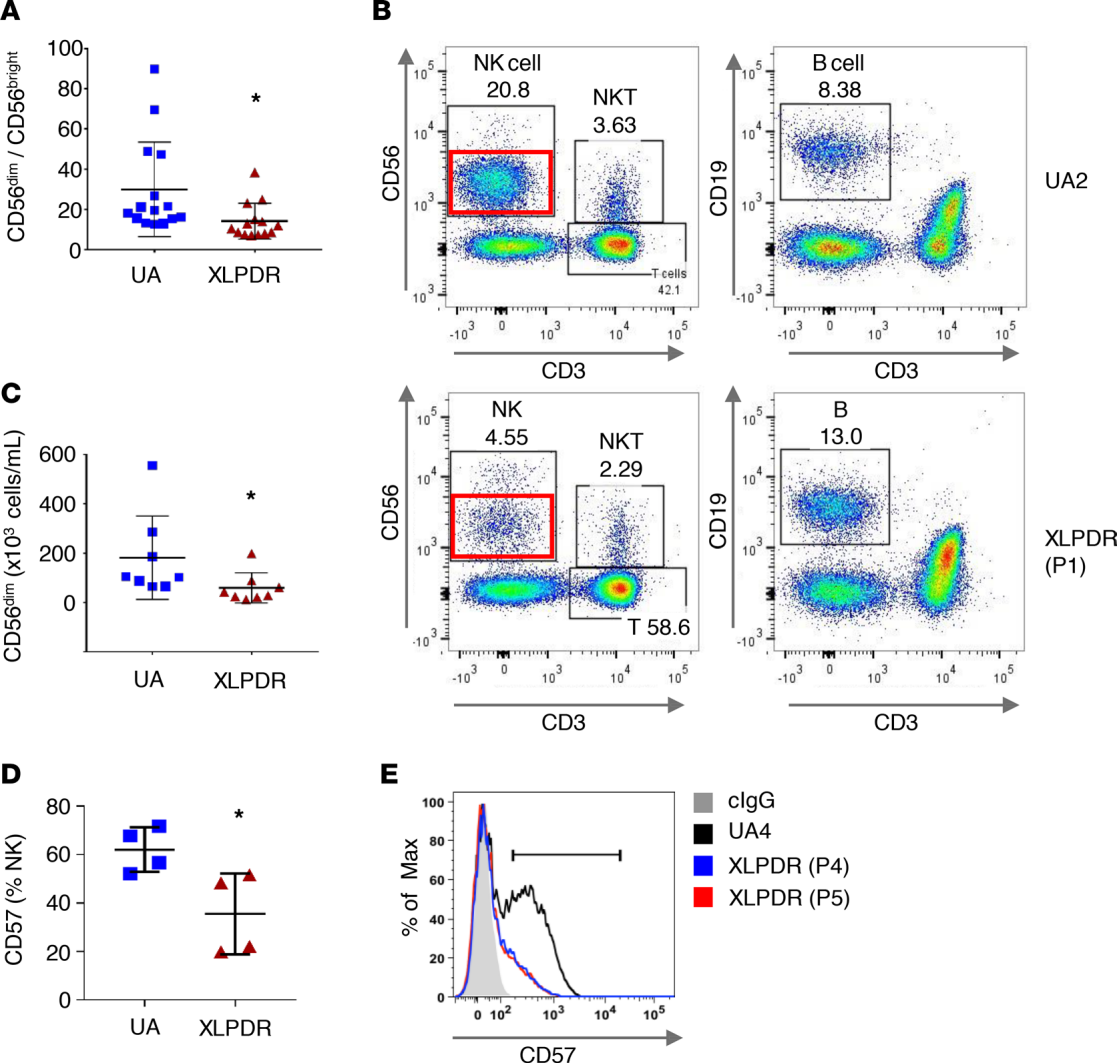
POLA1 deficiency affects NK cell maturation and direct cytotoxicity. (**A**) Flow cytometry quantification of the CD56^dim^/CD56^bright^ ratio in peripheral NK cells is significantly affected in XLPDR patients (P1–P5) compared with unaffected control (UA1–UA7, UA10, UA11). Horizontal bars represent the mean; error bars correspond to the SD; *P < 0.02 by Student’s 2-tailed *t* test. Data are the aggregate of 5 independent experiments. (**B**) Representative flow cytometry plots of white blood cell subpopulations in an unaffected individual (UA2) and an XLPDR patient (P1). CD56^dim^ (stage V) NK cell subsets are highlighted by red rectangles. (**C**) Flow cytometry–based quantification of stage V CD56^dim^ NK cells in unaffected individuals (UA4–UA7, U10, UA11) and XLPDR patients (P1–P5). Horizontal bars represent the mean; error bars correspond to the SD; *P < 0.04 by Student’s 1-tailed *t* test. Data are the aggregate of 2 independent experiments. (**D**) Flow cytometry–based quantification of stage VI CD57^+^ NK cells in unaffected individuals (UA1–UA4) and XLPDR patients (P1, P2, P4, P5). Horizontal bars represent the mean; error bars correspond to the SD; *P < 0.03 by Student’s 2-tailed *t* test. Data are the representative image of 3 independent experiments. (**E**) FACS analysis of CD56^+^CD3^–^ NK cells depicting representative CD57 surface expression in an XLPDR proband (P4, P5) compared with an unaffected control (UA4).

*MCM4* mutations have been reported to cause markedly reduced levels of mature stage V CD3^–^CD56^dim^ NK cells (14, 22). With this in mind we examined whether NK subpopulations were affected in patients with XLPDR. First, we noted that the CD56^dim^ (stage V) to CD56^bright^ (stage IV) ratio in patients with XLPDR was significantly reduced, indicating a relative reduction in stage V CD56^dim^ NK cells (Figure 3A). Direct quantification of this population (Figure 3B, red rectangle) indicated that XLPDR is associated with a decrease in the total number of circulating stage V CD56^dim^ NK cells (Figure 3C). Furthermore, CD57-expressing cells within this population, which represent terminally differentiated and highly cytotoxic NK cells, were also reduced in XLPDR (Figure 3, D and E). Therefore, XLPDR is also associated with impaired differentiation of NK cells, which appears quantitatively milder than described in IMD54 (14–17).

### POLA1 deficiency does not affect DNA recombination and repair.

**Figure 4:**
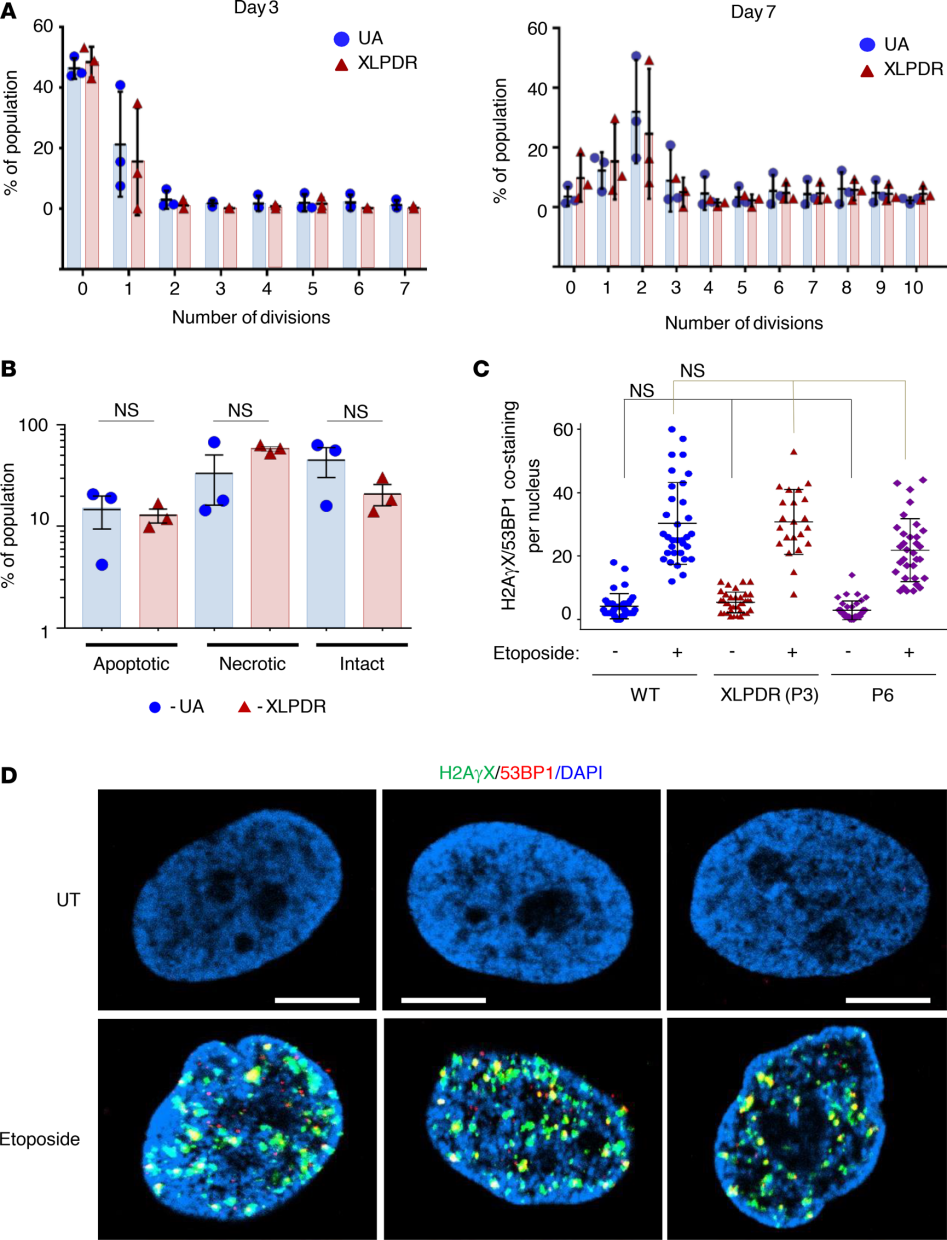
POLA1 does not affect cell cycle progression and DNA integrity. (**A**) Flow cytometry analysis of NK cell proliferation (based on CFSE dilution) was performed on days 3 and 7 in NK cells isolated from unaffected individuals (UA4, UA5, UA8) and from XLPDR probands (P1, P2, P3). Error bars represent the SEM. XLPDR and UA groups are not different at every division number (*P* > 0.05 by 2-way ANOVA). Data are representative of 2 independent experiments. (**B**) Flow cytometry analysis of apoptosis and necrosis (based on annexin V staining) was performed on day 14 in proliferating NK cells as above. Data are the result of 1 experiment. Black bars indicate the mean and error bars represent the SD. (*P* > 0.05 by 2-way ANOVA.) (**C**) Quantification of the number of DNA-damaging events per nucleus (colocalized H2AγX and 53BP1 foci) is presented. Each dot represents a cell in each group. Bars correspond to the group average, and error bars correspond to the SD. NS, nonsignificant when compared to its corresponding control (*P* > 0.05 by 2-way ANOVA). (**D**) Representative images of DNA damage foci assessed by immunofluorescence staining and confocal microscopy imaging for nuclear H2AγX and 53BP1. Dermal fibroblasts from an XLPDR patient that were rescued for POLA1 expression (P3 + POLA1) were compared to an isogenic control line (P3 + EV) and to fibroblasts from P6. Staining at baseline and after etoposide treatment (1 μM, 5 hours) is shown. Scale bar: 2 μm. Representative images were derived from 2 independent experiments.

MCM4-deficient patients’ fibroblasts and NK cells have reduced proliferative capacity, which is thought to mediate the lack of differentiation to stage V NK cells (14). Similar reduced proliferative capacity has been reported in patients with *GINS1* mutations (21). This stands in contrast with prior studies indicating that XLPDR-derived fibroblasts have normal proliferative capacity at baseline (10) and more recent studies in patients with novel *POLA1* mutations, which further confirm this finding (13). Thus, we examined the proliferative capacity of XLPDR-derived NK cells in vitro and found that these cells did not have impaired proliferation based on CFSE dilution analysis (Figure 4A). Moreover, the rates of apoptotic and necrotic cells during NK cell proliferation did not differ between patients with XLPDR and unaffected individuals (Figure 4B).

In addition to their role in DNA replication, the MCM and GINS complexes participate in maintaining genomic stability. Fibroblasts from patients with *GINS1* mutations have been found to have nuclear abnormalities and increased propensity to double-stranded DNA damage under conditions of pharmacological stress (21). Similarly, *MCM4* mutations are associated with increased susceptibility to double-stranded DNA breaks and aneuploidy (14). Thus, we analyzed whether *POLA1* deficiency is associated with increased susceptibility to DNA double-stranded breaks. Using H2AγX and 53BP1 as markers of DNA damage sites, dermal fibroblasts from P3 (XLPDR) and P6 (*POLA1* c.328G>A) displayed a similar number of damage foci as a control line, and this remained true even after introducing the DNA-damaging agent etoposide (Figure 4, C and D).

### Defective XLPDR NK cell cytotoxicity is associated with impaired cytotoxic granule mobilization.

**Figure 5:**
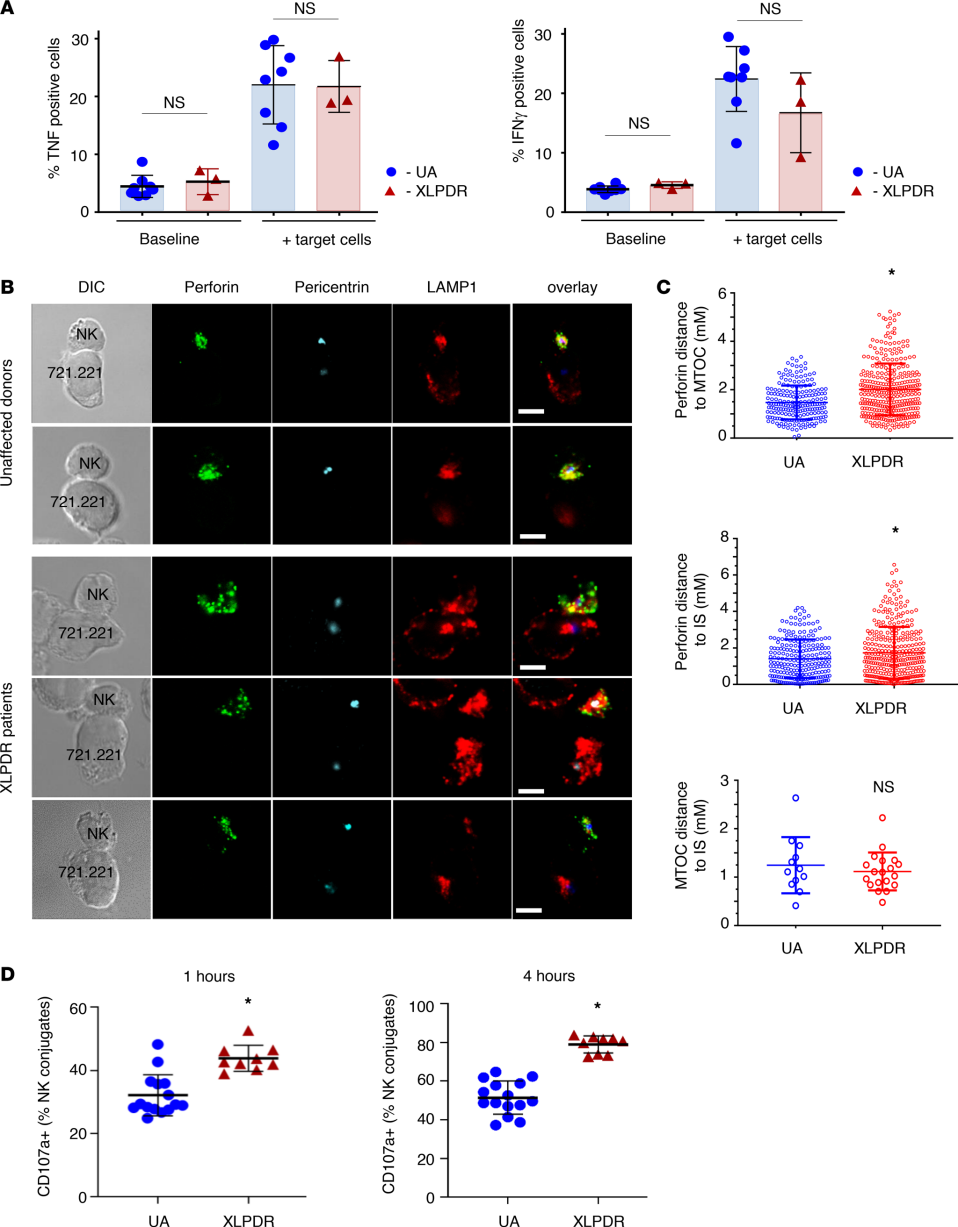
POLA1 deficiency affects polarization of lytic granules in activated NK cells. (**A**) Flow cytometry analysis of TNF (left) and IFN-γ (right) expression in NK cells, derived from unaffected control individuals (UA1–UA3) and XLPDR patients (P1, P2). Data are derived from 1 experiment; error bars correspond to the SD. NS, nonsignificant (*P* > 0.05, 2-way ANOVA). (**B**) Lytic granule and MTOC complex mobilization in NK cells was examined by immunofluorescence staining and confocal microscopy after activation with 721.221 target cells. Perforin (green), pericentrin (MTOC marker; blue), and LAMP1 (red) are depicted. The dashed lines indicate the position of the cell-cell contact site. Scale bars: 5 μm. Representative images of cells from healthy donors and patients with XLPDR are presented. (**C**) Quantification of confocal microscopy data from **B**. Distances from MTOC to perforin granules (top), perforin granules to the immunological synapse (center), and MTOC to the immunological synapse (bottom) are presented. Analysis was done using all available images from XLPDR NK cells (*n* = 19) and unaffected NK cells (*n* = 12). *P < 0.05; NS, not significant (*P* > 0.05), by 2-tailed Student’s *t* test. (**D**) LAMP1 degranulation in NK target conjugates. NK cells were derived from unaffected individuals (UA6, UA7, UA11, UA12) and XLPDR patients (P3–P5) and incubated for 1 or 4 hours with target 722.221 cells, and surface expression of CD107a was assessed by flow cytometry. Data are the representative image of 3 independent experiments. Horizontal bars represent the mean; error bars correspond to the SD; *P < 0.0001 by Student’s 2-tailed *t* test.

NK cell activation involves signal transduction events that have a number of consequences, including the induction of cytokine expression (23). Thus, we examined TNF and IFN-γ induction in these cells following engagement with target cells and found no significant differences between control and XLPDR patients in the number of TNF^+^ and IFN-γ^+^ NK cells (Figure 5A). Another event during NK cell activation is the polarization of the microtubule organizing complex (MTOC) and lytic granules toward the immunological synapse upon NK cell engagement of its cellular target (24, 25). Using immunofluorescence staining and confocal microscopy, we examined these critical events in primary NK cells derived from patients with XLPDR (Figure 5B). NK cells from unaffected individuals readily polarized with the MTOC moving toward the immunological synapse and forming tight lytic granule clusters (marked by perforin) near the point of cell-cell contact. NK cells isolated from patients with XLPDR also showed normal polarization of the MTOC complex toward the immunological synapse but displayed defective mobilization of lytic granules toward the MTOC and the immunological synapse (Figure 5, B and C). Next, we examined whether this defect in lytic granule polarization was associated with alterations in lytic granule fusion with the plasma membrane. This process normally results in the incorporation of LAMP1 on the cell surface, indicative of NK cell degranulation. This analysis indicated that surface LAMP1 staining was increased at 1 and 4 hours in NK cells from patients with XLPDR (Figure 5D). Thus, NK cell degranulation was not diminished in these cells but rather was inappropriately localized.

## Discussion

In this study we demonstrate that *POLA1* deficiency, induced experimentally or seen in patients with XLPDR, results in NK cell functional deficiency that can be traced to a defect in lytic granule mobilization. Furthermore, we identified partial deficiency of CD3^–^CD56^dim^ stage V NK cells in these patients and propose that the immunodeficiency associated with XLPDR may be in part the result of these alterations in the NK cell compartment.

Although NK cell development is affected in many syndromes of defective cellular immunity, few genetic causes of pure NK cell deficiency have been identified to date. These include autosomal dominant *GATA2* mutations; deficiencies of *MCM4*, *GINS1*, *RTEL1*, and *IRF8*; as well as the mutations in *CD16* that lead to a functional NK cell deficiency (26, 27). Based on the phenotypes seen in these disorders, NK cells appear to be important in lung immune defense because some of these patients develop recurrent lung infections and bronchiectasis, a cardinal phenotype also observed in XLPDR (Supplemental Table 2). Moreover, concurrent severe NK cell lymphopenia in patients with combined variable immunodeficiency is associated with more severe lung infections (18), further suggesting that NK cells play an important role in lung defense. In addition, many NK deficiency syndromes, including IMD54, are associated with predisposition to severe infections by viruses of the Herpes family (14–16, 18, 26), a feature not observed in XLPDR (Supplemental Table 2). This may reflect the fact that the numerical defect in NK cell populations is much milder in XLPDR compared with other NK cell deficiencies, such as IMD54 (14). Furthermore, because of alterations in nucleic acid metabolism, XLPDR is also associated with profound type I IFN activation (10), which has not been reported in other NK cell deficiencies and may be responsible for these differences. In fact, some of the unique aspects of XLPDR, such as its autoinflammatory manifestations (keratitis, urinary tract strictures, and gastrointestinal inflammation) are probably because of its associated interferonopathy and appear unrelated to NK cell dysregulation (10, 11).

The work further suggests that the NK cell phenotype in XLPDR may be linked to the effects of *POLA1* expression on *MCM4*. As shown here, cells derived from patients with XLPDR are partially deficient in *MCM4*, and interestingly, deficiency of either *POLA1* or *MCM4* in primary NK cells or NK cell lines results in a functional defect in NK cell cytotoxicity. This latter finding indicates that the NK cell defect in patients with *MCM4* hypomorphic mutations is not only quantitative, but also qualitative, and suggests possible noncanonical functions of Pol-α/primase and MCM and GINS complexes in NK cell cytotoxic function.

**Figure 6:**
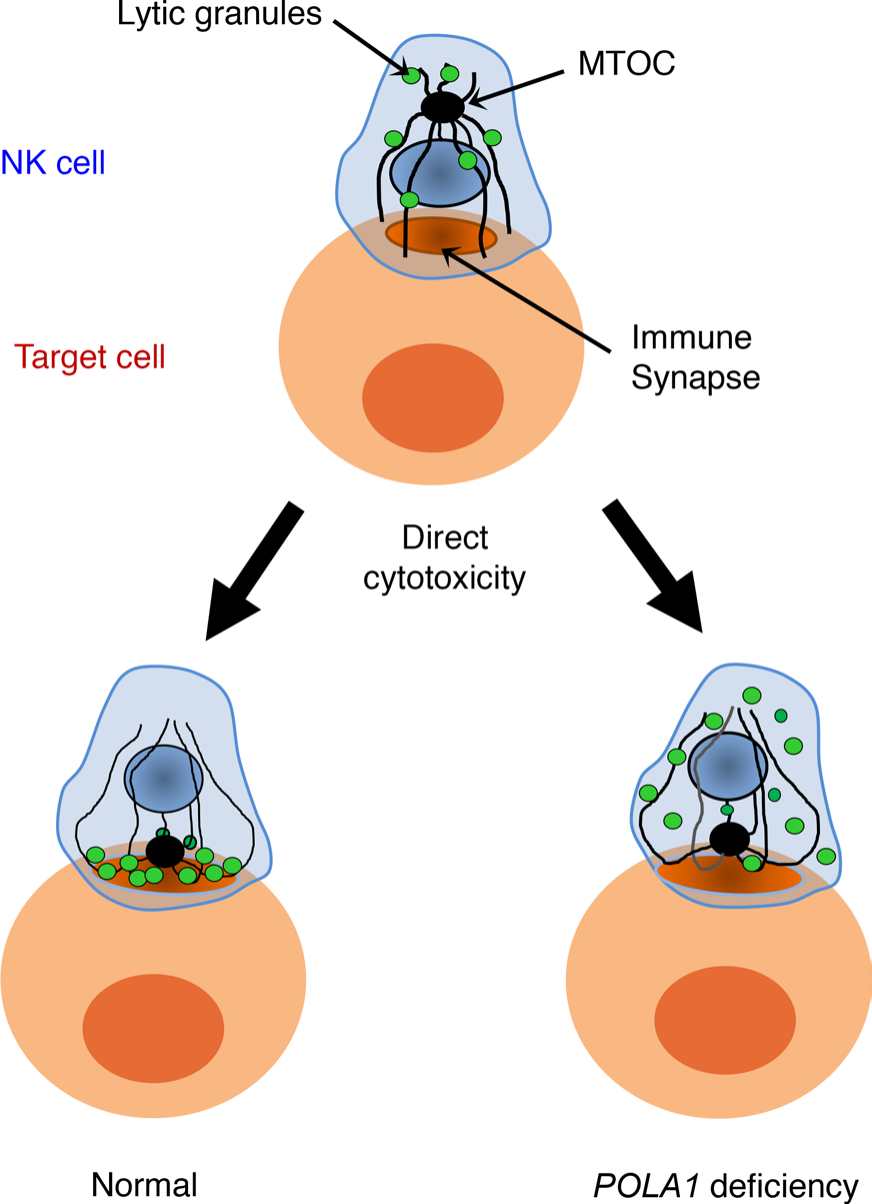
POLA1 deficiency affects cytotoxic granule polarization in NK cells.

Mechanistically, the defect in lytic activity that occurs upon *POLA1* deficiency can be traced to a specific defect in lytic granule mobilization in the context of direct cytotoxicity (Figure 6). As shown here, lytic granules fail to mobilize toward the immunological synapse, but based on LAMP1 degranulation experiments, the lytic granules ultimately fuse even at higher levels but in the wrong membrane domain. A similar phenotype of misdirected degranulation and poor cytotoxic activity has been reported upon NK cell defects in VASP, an actin cytoskeleton regulator (28).

Interestingly, the cytotoxicity defect is not seen during ADCC, suggesting a specific impairment of direct cytotoxicity, such as defective cellular activation during this process. In particular, altered NF-κB signaling, which is a known feature of XLPDR (10), is an attractive possibility because this can result in abnormal NK cell phenotypes. At present, altered NK cell function is known to develop in genetic syndromes involving genes in the NF-κB pathway in humans (29, 30). For example, several hypomorphic or loss-of-function mutations in NEMO (*IKKG*), IκBα (*NFKBIA*), p100 (*NFKB2*), or NIK (*MAP3K13*) are associated with immune phenotypes, including NK cell deficiency or impaired direct cytotoxicity with preserved ADCC function (31, 32). Intriguingly, patients with overactive *NFKB2*, responsible for the so-called noncanonical NF-κB pathway, may develop ectodermal dysplasia, a feature typical for XLPDR (10), as well as adrenal insufficiency, which is a feature of the MCM4 deficiency phenotype (14–16). Thus, constitutive activation of noncanonical NF-κB is a potential explanation for the linkage between POLA1 and NK cell dysfunction that deserves future investigation.

In addition to the functional defects shown here, XLPDR shares with IMD54 a reduction in the number of circulating stage V NK cells. MCM4 and GINS1 mutations are associated with significant growth delay, which is also seen in POLA1 mutations that lead to limited functional reserve (13), but not in XLPDR (9–11). Because MCM4 deficiency leads to reduced cell proliferation, the NK cell differentiation defect has been thought to result from this proliferation defect. However, prior work indicates that under unstressed conditions POLA1 deficiency does not lead to altered proliferation in fibroblasts (11), and the same is true for NK cell proliferation in vitro, as shown here. In fact, recent studies indicate that POLA1 deficiency leads to increased distance between origins of replication, and only under conditions of replicative stress, there is an overt proliferation defect (12). One possible explanation for the preserved proliferative capacity under baseline conditions is that Pol-α/primase function can be supported by the recently discovered PrimPol DNA polymerase (33). The short stature and intrauterine growth delay seen in patients with POLA1 mutations (other than XLPDR) suggest that under specific conditions, deficiency in Pol-α/primase can lead to cellular and clinical phenotypes that have greater overlap with IMD54 and *GINS1* mutations. As far as the explanation for the specific depletion of stage V and VI NK cells, it is possible that this is due to replicative stress that occurs in vivo that is not properly modeled by in vitro NK cell proliferation conditions. Alternatively, the defect in NK cell development may be due to effects on NK cell differentiation that are unrelated to cell proliferation. In this regard, other mutations in MCM complex components, such as *MCM5*, also impair cellular proliferation but do not affect the NK cell compartment (34). Thus, impaired cellular proliferation due to helicase defects is not sufficient to cause NK cell phenotypes. Similarly, mutations in the origin recognition complex resulting in Meier-Gorlin syndrome (34) lead to defects in replication and MCM complex loading on chromatin but are not associated with NK cell phenotypes in vivo. These observations support the notion that replication defects are not sufficient to produce NK cell defects and that MCM4 and POLA1 may affect NK cell function through additional mechanisms. Finally, it is worth noting that severe *POLA1* deficiency leads to hypogonadism, whereas MCM4 mutations lead to adrenal insufficiency, both states of reduced steroid hormone production. In both cases, the mechanisms for such endocrine defects are unclear, and the rarity of these syndromes makes it very difficult to understand the underlying pathophysiology.

Lack of animal models hampers progress in our understanding of the mechanisms for many of these conditions, including XLPDR, which is itself extremely rare in humans. For example, further molecular details regarding the role of MCM4 in NK cells are still elusive because this deficiency is hard to recapitulate in mouse models. Complete *MCM4* deficiency is lethal and most studies rely on a hypomorphic mutant (*MCM4^Chaos3^*), which demonstrates not only a decrease in NK cells but also alterations in other lymphocyte populations (35). Furthermore, the NK cell defect appears to be far too mild to reproduce the phenotype of patients. Development of a murine model of XLPDR that can recapitulate features of this syndrome would be a major advance for this field.

Altogether, we found that POLA1 deficiency, either resulting from naturally occurring mutations or as a result of experimental manipulations, impairs NK cell cytotoxicity. Moreover, we showed that this NK cell functional deficiency is due to a defect in lytic granule mobilization toward the immunological synapse. Finally, we provide evidence of a functional connection between the Pol-α/primase and the MCM complexes outside of the replication process, explaining the overlap in clinical features between IMD54 and XLPDR.

## Methods

### Cell culture and transfection.

All cell lines used in this study were tested for mycoplasma using a PCR-based method (24). This testing was performed upon obtaining the cells and every 8–12 weeks to confirm that all experiments were performed in uninfected cells. HEK293T cells (American Type Culture Collection, ATCC) were cultured in DMEM supplemented with 10% FBS and l-glutamine. NK92mi cells were grown in X-vivo medium (Lonza) with 10% human serum. YTS cells were cultured in IMDM supplemented with 12.5% FBS. Human B lymphoblastoid 721.221 cells and ovarian carcinoma SKOV-3 cells were cultured in complete RPMI 1640 medium (Gibco, Thermo Fisher Scientific). Patient-derived dermal fibroblasts were obtained after IRB approval and informed consent, as previously described (10). Fibroblasts were cultured in DMEM supplemented with 10% FBS. Etoposide (MilliporeSigma) was used as a 1-mM solution in DMSO. Poly(dA:dT) (1 mg/mL, InvivoGen) was transfected using LyoVec (InvivoGen) following the manufacturer’s instructions.

### Primary NK cell isolation.

Blood from patients with XLPDR was collected locally and shipped for analysis along with samples from control individuals. For other studies, normal NK cells were obtained from healthy donors recruited at the Department of Transfusion Medicine, NIH, with the donors’ informed consent, in accordance with the Declaration of Helsinki. Approximately 50 mL of peripheral blood was collected by venipuncture at room temperature in tubes containing sodium heparin. Peripheral blood mononuclear cells (PBMCs) were isolated from whole blood using the standard Ficoll-Paque method. NK cells were isolated from PBMCs using EasySep Human NK cell enrichment kits (STEMCELL Technologies) according to the manufacturer’s protocol. NK cells were cultured in X-vivo medium (Lonza) supplemented with 10% human serum and 100 U/mL of IL-2.

### RNA-Seq and gene expression data analysis.

The data set analyzed has been previously described (10) and deposited in GEO (accession number GSE72589). Briefly, RNA was extracted from dermal fibroblasts, including cells from an unaffected male (UA) and an XLPDR patient (X2) and 2 samples of fibroblasts from an unaffected donor and were transfected with either control siRNA or a duplex targeting POLA1 (siPOLA1). RNA-Seq library preparation, sequencing, and analysis have been described in detail (10).

### Bioinformatic analyses.

The GEO provides a public repository for high-throughput gene expression data, including tools to query and analyze data sets (36). We queried GEO profiles of human POLA1, sorting the results by subgroup effect. Those profiles with visually inconsistent expression levels of POLA1 across conditions were filtered out to keep those data sets with consistent expression levels within the same conditions and different levels among conditions. Genes corresponding to the top 200 profile neighbors of 11 resulting GEO data set records were collected (Supplemental Table 1, sheet 1). Consistently coexpressed profile neighbors were counted across data sets and assigned a score corresponding to the simple count. We limited consistently coexpressed genes to those that were profile neighbors in more than 3 data sets (45 genes, Supplemental Table 1, sheet 2). A query of the more rigorous precomputed coexpression database, COXPRESdb (37), revealed top-ranking coexpressed genes across animal species (top 200, Supplemental Table 1, sheet 3); 18 out of 45 of the consistently coexpressed genes are in the top 200 COXPRESdb list. Next, we ran Gene Ontology (GO) term enrichment analysis on the consistently coexpressed gene list from GEO data sets using PANTHER (version 13.1) overrepresentation test as compared with the reference human genome (38). We applied Fisher’s exact test with FDR multiple-test correction for GO-slim biological process annotations (PANTHER version 13.1), keeping reporting results with FDR less than 0.05 (Supplemental Table 1, sheet 5). Using the more complete coexpression gene list from COXPRESdb, we performed functional clustering of all GO Direct terms (BP, CC, and MF) with the Database for Annotation, Visualization and Integrated Discovery server (39). Restricting terms to an EASE enrichment threshold of 0.01 and clustering with medium stringency produced 5 clusters (Supplemental Table 1, sheet 6) with similar terms as the more limited data set.

### Antibodies.

Cell lysate preparation, immunoprecipitation, and immunoblotting were performed as previously described (40). The following antibodies were used for Western blot studies: β-actin (MilliporeSigma, A5441), MCM4 (Abcam, ab4459), POLA1 (Santa Cruz Biotechnology, sc-5921), and PRIM2 (Bethyl, A305-274A). The following antibodies were used for immunostaining (including both flow cytometry and confocal microscopy): CD56 (BioLegend, HCD56), CD57 (BioLegend, HCD57), CD16 (BioLegend, 3G8), CD69 (BioLegend, FN50), perforin (BioLegend, dG9), LAMP1 (BioLegend, H4A3), pericentrin (Abcam, ab4448), anti-HER2 antibody (a gift from Q. Li, National Cancer Institute/Center for Cancer Research [NCI/CCR], Bethesda, Maryland, USA), H2AγX (MilliporeSigma, 05-636), and 53BP1 (Cell Signaling Technology, 4937).

### RNA interference.

RNA duplexes used for siRNA were purchased from MilliporeSigma and included the following: control nontarget siRNA (SIC002), siPOLA1 1 (SASI_Hs01_00138924) and siPOLA1 2 (SASI_Hs01_00138925), and MCM4 1 (SASI_Hs01_00218872), and MCM4 2 (SASI_Hs01_00218873). For siRNA transfection of fibroblasts, the DharmaFECT transfection system (DharmaFECT 4, T-2004-03, Dharmacon) was used according to the manufacturer’s instructions. Transfection of siRNA into primary NK cells (isolated ex vivo), as well as NK92mi and YTS cells, was performed by nucleofection. Two million cells were suspended in the NK cell nucleofection kit (ex vivo NK cells) or cell line nucleofector kit V (YTS cells) or kit R (NK92mi cells). The nucleofection was performed using Nucleofector II (Lonza) with program U-001 (for NK cells) or O-017 (for YTS and NK92mi cells). NK and NK92mi cells were analyzed 48 hours after the nucleofection. For YTS cells, a second nucleofection was performed 24 hours after the first nucleofection, and the cells were analyzed 48 hours later. POLA1 or MCM4 expression levels were monitored at the protein level by immunoblotting or by flow cytometry.

### Cytotoxicity assays.

NK cell cytotoxicity was evaluated by lanthanide (Europium, Eu) fluorescence assay. Briefly, target cells were labeled with BATDA (PerkinElmer) for 30 minutes at 37°C in the complete medium. Labeled cells were transferred to 96-well polystyrene plates (U-bottom, Nunc), mixed with NK cells at different E/T ratios, and incubated for 2 hours at 37°C. For ADCC, NK cells were incubated with SKOV-3 target cells in the presence of 50 ng/mL of either anti-HER2 antibody or control IgG. After cytolysis, the cell supernatant, containing the released TDA ligand, was added to the Eu solution to generate the fluorescent Eu-TDA chelate. The fluorescence of Eu-TDA was measured using EnSpire multimode plate reader (PerkinElmer). The amount of released TDA ligand in cell supernatants was regarded as the experimental TDA release. Total TDA release was measured after complete lysis of target cells by 1% NP-40. Lysis percentage was calculated using the following equation: (experimental TDA release – spontaneously released TDA) / (total TDA release – spontaneously released TDA).

### Flow cytometry.

For the analysis of the cell surface receptors, NK cells were stained with the antibodies indicated in the *Antibodies* section and analyzed by flow cytometry. Compensation controls were set with the same antibodies (Abs) used to stain the cells and the CompBead Plus kit (BD 560497). For analysis of total protein levels of LAMP1, NK cells (3 × 10^5^) were fixed and permeabilized with Cytofix/Cytoperm buffer (BD 554722) and then stained with anti–LAMP1-FITC Ab. The data acquisition and analysis were done using FACSCalibur or LSRII cytometers (BD Biosciences) and FlowJo software (Tree Star, Inc., v. 10).

### Proliferation and cell death assays.

To enable tracking of proliferation, NK cells used for proliferation analysis were prestained with CFSE, and stain was verified using analysis of a sample by flow cytometry. Briefly, CFSE Proliferation Dye (eBioscience, 65-0850-85) was added to the cells, and they were incubated for 10 minutes at 37°C protected from light. Complete RPMI 1640 was added and the sample was incubated for an additional 10 minutes in the dark. The cells were then washed twice in RPMI before being added to the culture.

After isolation and CFSE staining, NK cells were placed into RPMI 1640 (Gibco, Thermo Fisher Scientific) with Pen-Strep (Corning), glutamine (Corning), sodium pyruvate (Corning), MEM nonessential amino acids (Corning), and 20% FBS (Atlanta Biologicals) supplemented with IL-2 (Peprotech). NK cells were maintained at approximately 0.5-1e6 NK cells/mL. For proliferation and cell death analysis, K562 cells genetically engineered to express membrane-bound IL-21 (3) were irradiated and placed in culture at a 2:1 K562/NK cells ratio. The culture was then evaluated for growth at different time points to determine the extent of growth. For proliferation analysis, the culture was pipetted to fully resuspend the cells, and a sample was removed for analysis. Cells were washed twice with 2% FBS in PBS and then stained with APC mouse anti–human CD56 (BioLegend, HCD56) for 1 hour at 4°C. The samples were then washed twice with 2% FBS in PBS and analyzed on a BD FACSCanto II flow cytometer. Cell proliferation analysis was done using the included function in FlowJo software (Tree Star, Inc., version 8.8.7).

### Apoptosis and necrosis analysis.

After the CFSE stain had become undetectable (day 14), staining for cell death was completed. Briefly, a Pacific Blue Annexin V Apoptosis Detection Kit with 7-AAD (BioLegend, 640926) was used. Briefly, samples were first stained per normal method with APC anti-CD56 antibody. Cells were then washed twice with 2% FBS in PBS and then resuspended in Annexin V Binding Buffer and Pacific Blue Annexin V was added. The samples were then incubated at room temperature in the dark for 15 minutes before being washed twice with Annexin V Binding Buffer. 7-AAD staining solution was added to each tube 15 minutes before it was analyzed by flow cytometry. Unless otherwise noted, cells were maintained at 4°C or on ice throughout this cell death staining protocol.

### Microscopy and image analysis.

NK cells were mixed with 721.221 target cells at a 1:1 ratio for 20 minutes at 37°C in X-vivo medium, followed by adherence to Excell Adhesion slides (EMS) for 10 minutes at 37°C. Cells were fixed and permeabilized with Cytofix/Cytoperm buffer with 0.1% Triton X-100 and blocked with 1% BSA. The cells were stained with anti-LAMP1 Ab followed by Alexa Fluor 647–conjugated IgG1-specific anti-mouse Ab (Life Technologies, A21236), stained with antipericentrin followed by Alexa Fluor 568–conjugated anti-rabbit Ab ((Life Technologies, A10042), blocked with 5% normal mouse serum, and then stained with Alexa Fluor 488–conjugated anti-perforin Ab (BioLegend, dG9). Cells, mounted in ProLong Gold medium (Thermo Fisher Scientific), were visualized by a Zeiss LSM710 Axiovert 200M laser-scanning confocal microscope at room temperature. The images were obtained using ×63 Zeiss Plan-Apochromat objective and Zeiss Zen 2012 software. Images shown represent a single optical section. The granule distances to the immunological synapse and MTOC were estimated based on a previously described method (24). To measure the distance between the MTOC and granules, the (*x*, *y*) coordinates of the MTOC and the granules (or the entire region outlined by lytic granules, if the centroids of individual granules could not be determined) in the plane of MTOC were obtained, followed by calculation of the length of the shortest line connecting the MTOC and granule centroids with Imaris software (v. 9.1.2, Bitplane). To determine the MTOC or granule distance to the immunological synapse, lines representing the shortest distance connecting the MTOC or granule centroids with the cell-cell contact site (determined from differential interference contrast images) were created using Imaris software.

### Whole-blood RNA analysis.

Blood samples from P6 (patient with *POLA1* mutation) and 3 healthy controls were collected in triplicate in PAX gene tubes (762165, PreAnalytix) at San Antonio Military Medical Center. Samples were processed together on the same day of sample collection. mRNA was isolated according to the manufacturer’s instructions and analyzed by quantitative real-time reverse transcription PCR (qRT-PCR).

### qRT-PCR.

RNA was extracted using TRIzol (Invitrogen, Thermo Fisher Scientific) according to the manufacturer’s instructions. Total RNA (3 μg) was used for cDNA synthesis utilizing the Superscript III strand synthesis system (Invitrogen, Thermo Fisher Scientific). qRT-PCR was performed using a Mastercycler (Eppendorf) following the manufacturer’s recommendations. SYBR Green–based detection (Invitrogen, Thermo Fisher Scientific) was employed using gene-specific primers noted in Table 4. Experiments were performed in duplicate, data were normalized to housekeeping genes (*ACTB*, *GAPDH*, 18S rRNAs, 28S rRNA), and the relative abundance of transcripts was calculated by the comparative ΔΔCt method.

### Statistics.

In all dot graphs, the mean is presented, and the error bars correspond to the SD. In all bar graphs, columns represent the mean, and the error bars correspond to the SEM. Statistical comparisons between 2 groups were performed using 1- or 2-tailed heteroscedastic Student’s t test. For multigroup comparisons, 2-way ANOVA analysis was performed. Statistical analysis of image quantification data was performed using the integrated ANOVA function of Imaris software (v. 9.1.2, Bitplane). A *P* value less than 0.05 was considered significant.

### Study approval.

Immunological analyses of affected patients and family members were performed after obtaining informed consent. All the studies were reviewed and approved by the IRB at University of Texas Southwestern and collaborating institutions.

## Author contributions

PS and EB designed the study. PS and AL performed most of the cellular and biochemical experiments. EB oversaw cellular and biochemical studies. KMW, KK, BO, AGK, and MP performed the NK analyses. PS, DDB, and EB oversaw NK cell acquisition and interpreted the data. ARZ recruited XLPDR subjects. LSD, LR, and EG coordinated clinical studies. LNK and NVG performed bioinformatic analyses. AR and QC performed selected biochemical experiments. PS and EB wrote the manuscript.

## Supplementary Material

Supplemental data

Supplemental Data Set 1

## Figures and Tables

**Table 4:**
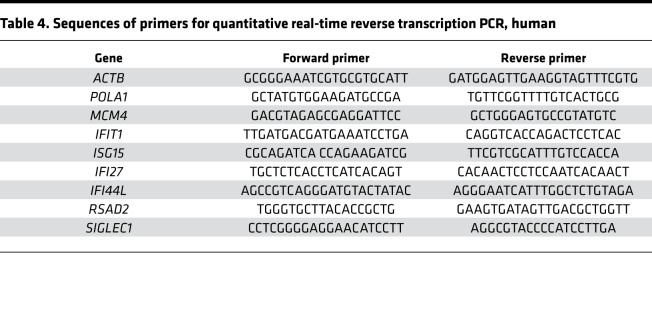
Sequences of primers for quantitative real-time reverse transcription PCR, human

**Table 3:**
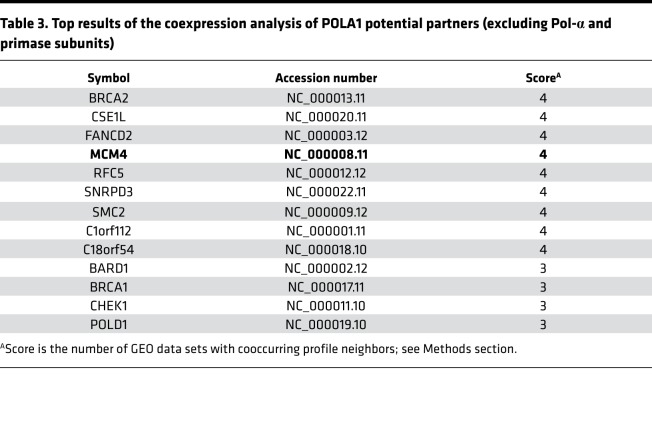
Top results of the coexpression analysis of POLA1 potential partners (excluding Pol-α and primase subunits)

**Table 2:**
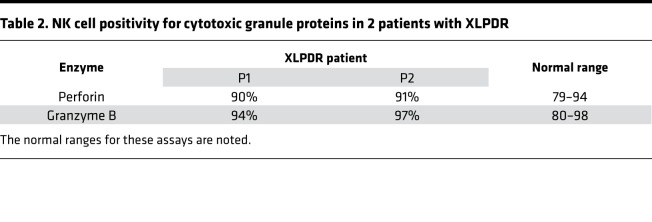
NK cell positivity for cytotoxic granule proteins in 2 patients with XLPDR

**Table 1:**
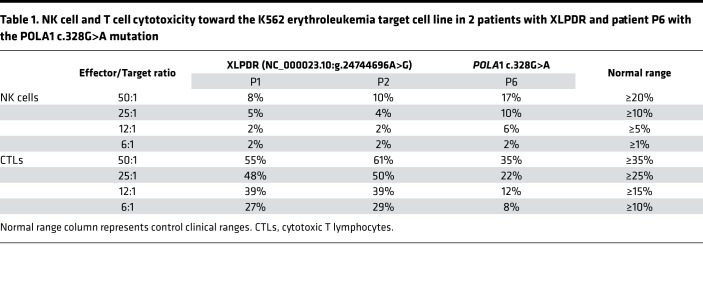
NK cell and T cell cytotoxicity toward the K562 erythroleukemia target cell line in 2 patients with XLPDR and patient P6 with the POLA1 c.328G>A mutation

## Version 1 11/01/2019

Electronic publication
