# Supplementary material for: NK cell defects in X-linked pigmentary reticulate disorder
Source: JCI Insight. 2019 Nov 1;4(21):e125688. doi: 10.1172/jci.insight.125688 (PMC6948767; doi:10.1172/jci.insight.125688)
Supplement: Supplemental data [file jciinsight-4-125688-s079.pdf]

Supplemental Figure 1

A

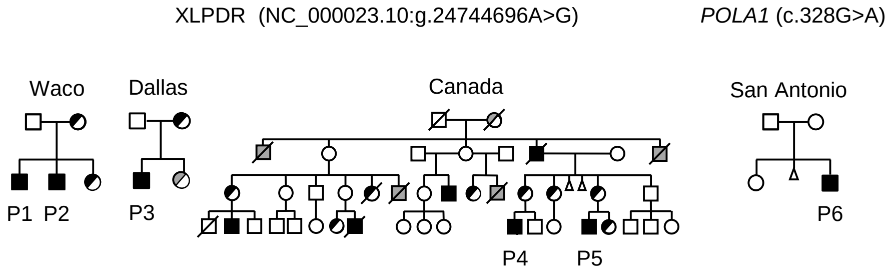

B

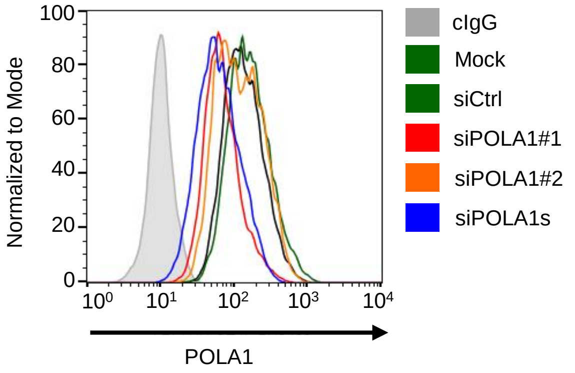

C

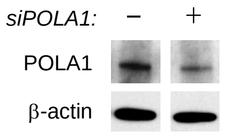

D

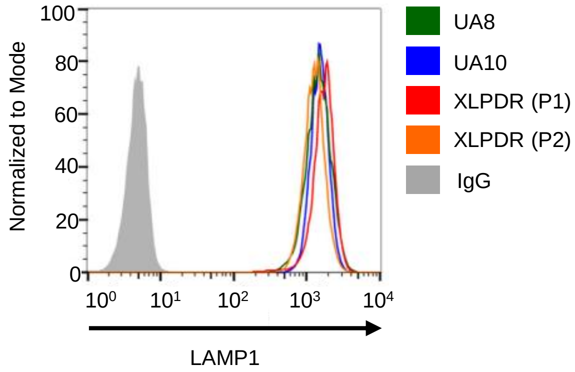

Supplemental Figure 2

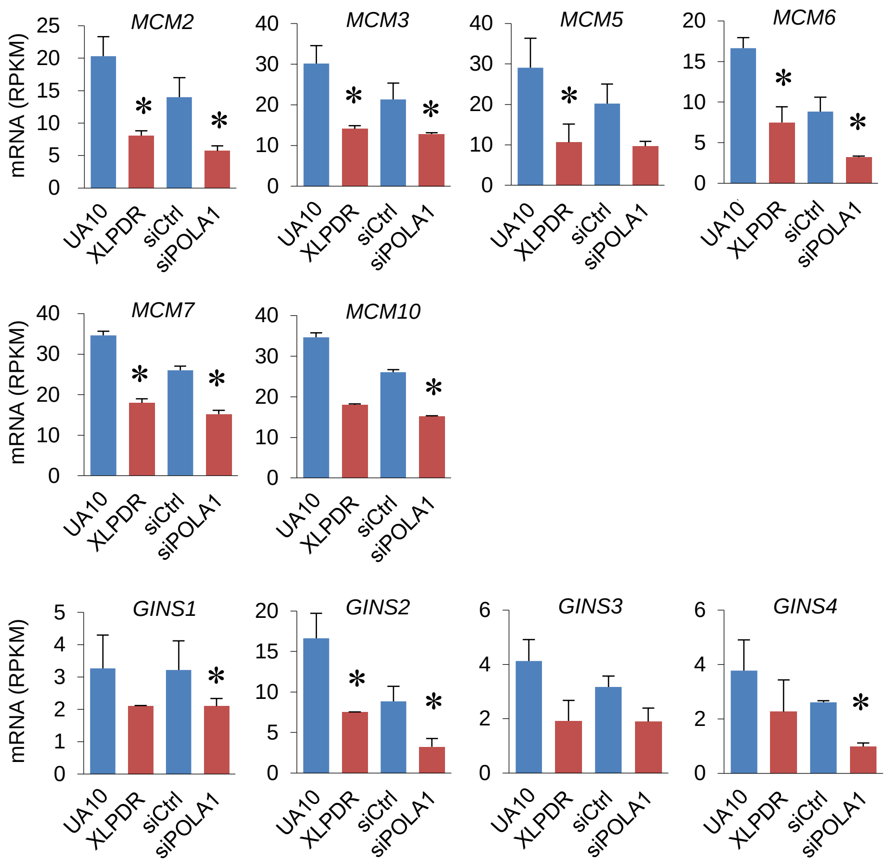

Supplemental Figure 3

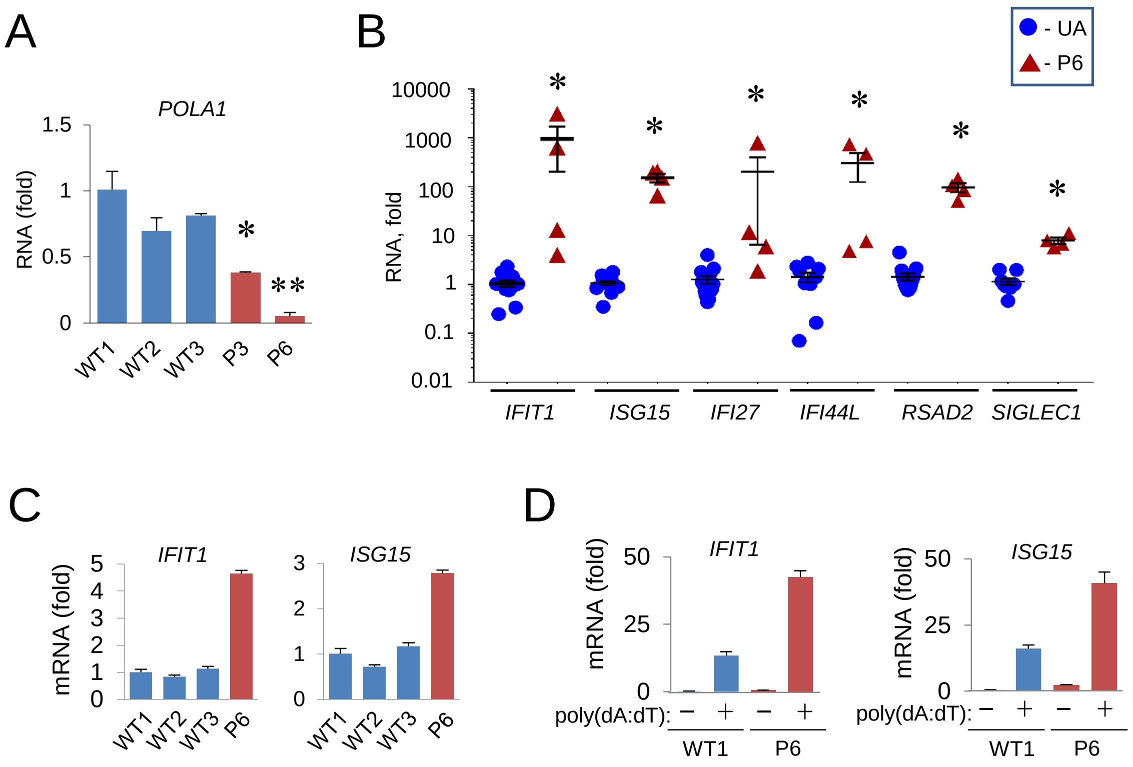

Supplemental Figure 4

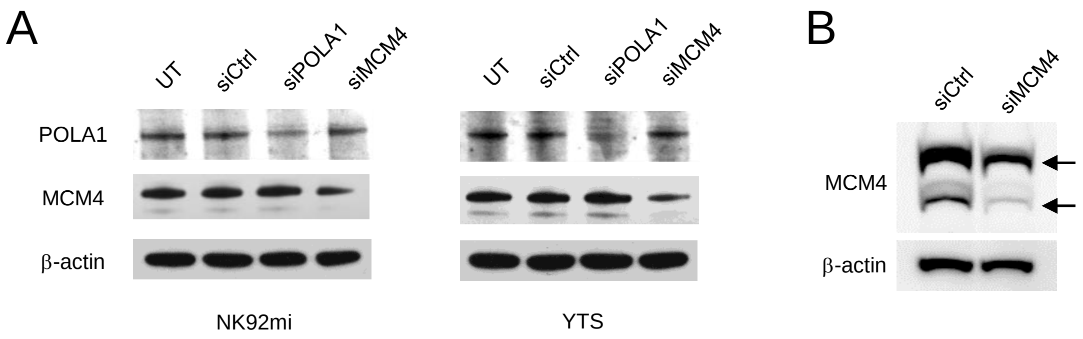

Supplemental Figure 1. POLA1 deficiency in NK cells.

A. Pedigrees of 3 previously reported XLPDR families that include the probands examined here (P1-P5); also shown is a family with a novel *POLA1* mutation (c.328G>A, P6). Place of origin is indicated for each family pedigree; affected individuals are marked in black, carriers in half blacked out symbols. Presumed cases and carriers are noted in grey.

B. Expression of POLA1 (corresponding to Figure 1D) was assessed by flow cytometry in primary NK cells after treatment with the indicated siRNA. Control IgG was used as a negative control; mock sample corresponds to untransfected primary NK cells.

C. POLA1 expression was determined by immunoblotting of protein lysate from NK92mi cells after siPOLA1 transfection (corresponding to Figure 1E). β-Actin serves as a loading control.

D. Expression of LAMP1 was assessed by flow cytometry in primary NK cells from unaffected controls (UA8, UA10) and XLPDR patients (P1, P2). Non-specific control antibody (IgG) was used as a negative control.

Supplemental Figure 2. Expression of selected components of the MCM and GINS complexes as determined by RNA-seq analysis. Samples are the same as in Figure 2A (UA10 vs XLPDR (P2, P3)) and Figure 2B (UA10 derived fibroblasts treated with siControl or siPOLA1). Bars represent the mean; error bars represent the S.E.M. *, $P<0.05$ by Student's 1-tailed *t* test. Data are the average of two or more independent experiments.

Supplemental Figure 3. Immunological characterization of the *POLA1* c.328G>A patient.

A. *POLA1* mRNA expression was assessed by qRT-PCR analysis in dermal fibroblasts from three unaffected male donors (WT), an XLPDR patient (P3), and the *POLA1* c.328G>A proband (P6). Bars represent the mean, and error bars the S.E.M. *, $P<0.05$; **, $P<0.001$ by two-way ANOVA test comparing P3 or P6 against WT samples. Data are the average of two experiments.

B. Transcript levels for interferon-stimulated genes were determined by qRT-PCR in whole blood-derived RNA from P6, and unaffected 'travel' controls including the proband's parents (UA14, UA15) and an unrelated male control (UA8). Transcript abundance is displayed as the fold rate compared to the same reference control sample. Black horizontal bar depicts the mean value; error bars correspond to the standard deviation. *, $P<0.05$ by Student's 1-tailed *t* test. Data are the aggregate results of two experiments.

C. Baseline *IFIT1* and *ISG15* mRNA expression by qRT-PCR in dermal fibroblasts derived from three unaffected males (WT) and P6. Data are representative of 2 independent experiments. Error bars represent the S.E.M.

D. *IFIT1* and *ISG15* mRNA expression in response to poly(dA:dT) stimulation (1 µg/mL, 16 hrs) was determined by qRT-PCR in dermal fibroblasts derived from unaffected male (WT1) and P6. Data are representative of 2 independent experiments. Error bars represent the S.E.M.

Supplemental Figure 4. Expression of MCM4 analyzed in different cell lines after siRNA treatment.

A. Cell lines NK92mi (left) and YTS (right) were transfected with the indicated siRNA, and after 48h of culture, cell lysates were prepared and assessed for POLA1 and MCM4 expression by immunoblotting. β-Actin serves as a loading control. This experiment corresponds to Figure 2D.

B. 293HEK was transfected with anti-MCM4 siRNA and expression of MCM4 was analyzed by immunoblotting. Arrows highlights two bands recognized by anti-MCM4 antibody. β-Actin is presented as a loading control. The image is representative of two independent experiments.
